# Mosaicking Opportunistically Acquired Very High-Resolution Helicopter-Borne Images over Drifting Sea Ice Using COTS Sensors

**DOI:** 10.3390/s19051251

**Published:** 2019-03-12

**Authors:** Chang-Uk Hyun, Joo-Hong Kim, Hyangsun Han, Hyun-cheol Kim

**Affiliations:** Unit of Arctic Sea-Ice Prediction, Korea Polar Research Institute, KIOST, Incheon 21990, Korea; chyun@kopri.re.kr (C.-U.H.); joo-hong.kim@kopri.re.kr (J.-H.K.); hyangsun@kopri.re.kr (H.H.)

**Keywords:** helicopter-borne imaging, very high-resolution, sea ice drift, commercial off-the-shelf sensor, time interpolation

## Abstract

Observing sea ice by very high-resolution (VHR) images not only improves the quality of lower-resolution remote sensing products (e.g., sea ice concentration, distribution of melt ponds and pressure ridges, sea ice surface roughness, etc.) by providing details on the ground truth of sea ice, but also assists sea ice fieldwork. In this study, two fieldwork-based methods are proposed, one for the practical acquisition of VHR images over drifting Arctic sea ice using low-cost commercial off-the-shelf (COTS) sensors equipped on a helicopter, and the other for quantifying the compensating effect from continuously drifting sea ice that reduces geolocation uncertainty in the image mosaicking procedure. The drifting trajectory of the target ice was yielded from that recorded by an icebreaker that was tightly anchored to the floe and was then used to reversely compensate the locations of acquired VHR images. After applying the compensation, three-dimensional geolocation errors of the VHR images were decreased by 79.3% and 24.2% for two pre-defined image groups, respectively. The enhanced accuracy of the imaging locations was affected by imaging duration causing variable drifting distances of individual images. Further applicability of the mosaicked VHR image was discussed by comparing it with a TerraSAR-X synthetic aperture radar image containing the target ice, suggesting that the proposed methods can be used for precise comparison with satellite remote sensing products.

## 1. Introduction

Observing the state and variation of properties of sea ice by satellite remote sensing is crucial for monitoring the effects of a changing climate. Extensive techniques have been applied to extract various physical properties of sea ice (e.g., distribution, concentration, thickness, roughness, surface composition, etc.) from remote sensing capabilities for multi-temporal data acquisition covering the entire polar regions. To validate those sea ice properties retrieved from satellite imageries, very high-resolution (VHR) on-site images acquired by imaging instruments on various platforms (airplane, helicopter, ship, etc.) have been used as the ground truth for sea ice concentration [1,2,3,4], topography [5,6], and surface composition [4,7,8].

Satellite, airborne and terrestrial lidars have also been used to provide the sea ice surface properties, such as sea ice surface types and surface topography, as reliable day and night measurement techniques through thin clouds [9]. The sea ice surface types varying from snow to bare ice, thus bringing the change of albedo [10], and their fractions have been validated using optical remote sensing images [9]. The surface topography of sea ice from terrestrial lidar point cloud has supported to understand the processes of seasonal evolution of sea ice [11,12]. Those lidar remote sensing-based sea ice properties can be validated using higher-resolution images by detailed discrimination of sea ice surface types and can be compared with the topographic features or the point densities over different features generated by multi-view VHR images [13], respectively.

VHR images are also useful for assisting fieldwork by recording the detailed sea ice condition or the locations of on-site measurements during the fieldwork period (e.g., [14,15]). Additionally, VHR images can be used independently for figuring out small-scale topographic features of sea ice [16,17,18] and extracting pancake ice floe size [19,20] without comparison to satellite remote sensing data.

On-site acquisition of VHR sea ice images can be implemented by various remote sensing platforms such as airplane, ship, helicopter, unmanned aerial vehicle (UAV), and so on. These platforms help image acquisition at lower altitude; therefore, optical images that are less affected by clouds can be obtained.

Airplanes have been used for image acquisition over a relatively wider area [7] and enable the employment of multiple sensors (e.g., laser altimeter, radar, and thermal sensor are equipped in the airplane for Operation IceBridge [14,18,21]). However, some drawbacks exist, e.g., the higher operational cost and the limited distance of operation, which is restricted by the location of airports or runways, compared to a ship-borne camera, UAV or helicopter carried onboard an icebreaker.

Ship-borne imaging sensors have been installed on icebreakers cruising polar oceans, e.g., [1,8,20,22]. The capability of long-trajectory data acquisition was considered as a merit of this platform, and enabled measurements of various sea ice characteristics such as concentration, type, floe size, and deformation. However, geometric distortion can be magnified in distant parts of image products due to oblique viewing geometry [22]. The limited areal range of image acquisition, i.e., within hundreds of meters of the icebreaker [22], can possibly lead to bias in the observed properties of sea ice within confined areas in situations of favorable conditions (thin and level ice) to icebreaker sailing. In case of measuring the sea ice concentration, underestimating bias can occur during summer.

Recently, UAV-based image acquisition has been applied to sea ice observation (e.g., [23,24]) due to the relatively low-cost and the ability to acquire VHR images with simple preparation and a short preparatory period. However, limited flight distance and less reliability in harsh environments, i.e., strong wind, low temperature and high humidity in polar oceans, has prevented regularized and extensive applications until now.

Helicopters are carried onboard icebreakers for logistics and scientific experiments (e.g., [2,4,5,6]). Helicopter-borne image acquisition is more reliable compared to UAV because of the enhanced endurance of helicopters, which allows them to withstand harsher environments, and their verified stability for long-term usage. Helicopters also have more flexibility for the attachment of multiple and heavier sensors, compared to airplanes. These advantages have allowed the measurement of sea ice thickness with electromagnetic (EM) induction sounding [25,26,27,28,29,30]. Occasionally, their primary purpose onboard icebreakers can be confined to logistics, not for scientific observation [31].

To validate the sea ice characteristics including surface topography, floe size distribution, ridge concentration, melt pond fraction, etc., aerial ground truth data (i.e., mosaicked VHR images) can be used because the mosaicked images have enough coverage and sufficiently fine spatial resolution for the validation purposes. However, continuous sea ice drift occurring during image acquisition causes distorted imaging locations with time, which eventually becomes an obstacle for precise image mosaicking and geographic matching with the lower-resolution remote sensing products acquired over larger areas at once. Moreover, it is not possible to select static ground-control points for conventional georeferencing and quality assessment. Previous studies have incorporated sea ice drift correction for the reconstruction of surface topography using aerial photographs from assembled stereo imaging sensor packages [5] or the comparisons between different remote sensing datasets, for example, satellite SAR images, helicopter-borne snow and ice thickness measurements using EM, sea ice roughness from helicopter-borne laser altimeter, and freeboard from airborne laser scanner [32,33].

This study aims to propose methods for the practical and cost-effective acquisition of helicopter-borne VHR images over drifting Arctic sea ice using commercial off-the-shelf (COTS) sensors equipped on a helicopter, carried onboard an icebreaker, and for the compensation of the effect from continuous sea ice drift from each opportunistically acquired image to reduce geolocation uncertainty during the image mosaicking procedure. The applicability of the mosaicked VHR image was discussed by direct comparison with a TerraSAR-X (TSX) synthetic aperture radar (SAR) satellite image including overlapped region.

## 2. Description of Study Area 

**Figure 1:**
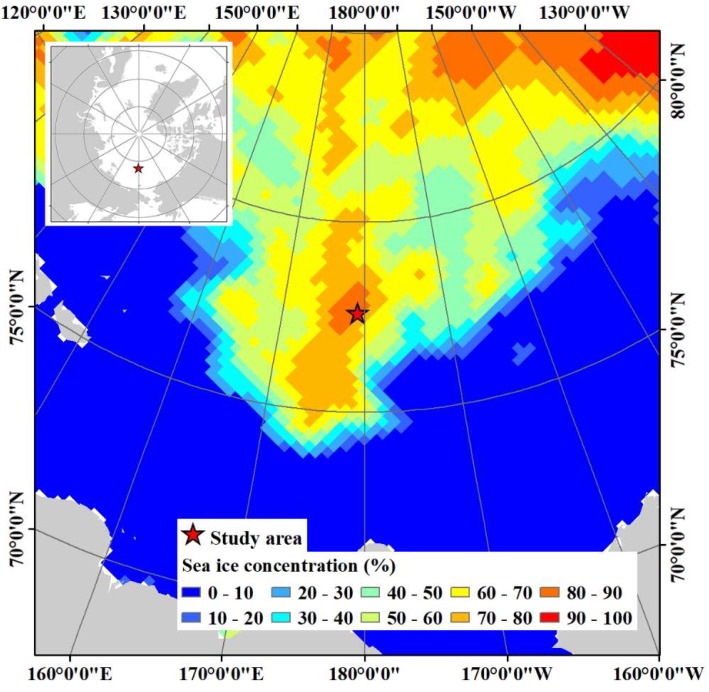
Location of our study area and surrounding sea ice condition on August 14, 2017 retrieved from the daily sea ice concentration data from Special Sensor Microwave Imager/Sounder (SSMIS) passive microwave data [34].

**Figure 2:**
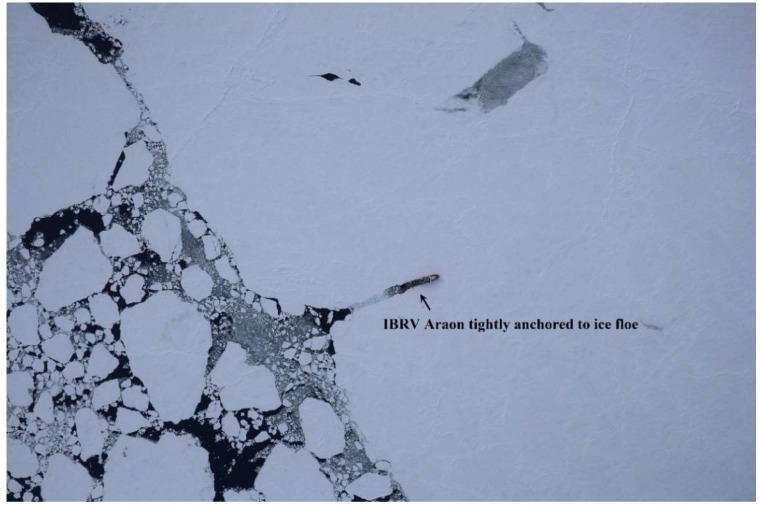
Aerial view of the sea ice floes where the field investigation was carried out on 13–15 August 2017, with the support of the icebreaking research vessel (IBRV) Araon, which was firmly anchored to the main ice. The photograph was taken by helicopter at an altitude of about 2285 m.

The study area is located in the Arctic Ocean, northwest of the Chukchi Sea around 77°36′ N/179°20′ E (Figure 1), where a field investigation was carried out on sea ice with the support of the Korean icebreaking research vessel (IBRV) Araon during 13–15 August 2017. Sea ice conditions on 14 August within the field investigation period were evaluated as ranging between 80% and 90% sea ice concentration from the daily data of the National Snow and Ice Data Center (NSIDC) using NASA Team algorithm with a cell size of 25 km [34]. During the field investigation on sea ice, the IBRV Araon was firmly anchored to a conglomerate of ice floes (hereafter, denoted as the main ice) by penetrating inside the ice floe (Figure 2).

## 3. Materials and Methods

Procedures of the VHR image acquisition, mosaicking, and accuracy assessment consist of: (i) helicopter-borne image acquisition with GPS logging; (ii) compensating for the effect from continuous sea ice drift using a time interpolated trajectory of the IBRV Araon; (iii) mosaicking the VHR images and error estimation; and (iv) testing the feasibility of the mosaicked VHR image. Detailed processes in each step are described in following sections.

### 3.1. Installation of Imaging Equipment on Helicopter

Two helicopters were carried onboard the IBRV Araon during the expedition. A commercial digital camera—a Canon M6 with 22 mm lens—was attached to the bottom of a BELL 206L-3 helicopter, with nadir viewing geometry. A GPS logger—a VBOX Sport (RACELOGIC, Buckingham, UK)—capable of 20 Hz logging (i.e., temporal resolution of 0.05 s) was placed inside the cockpit, 0.8 m higher than the position of the digital camera. Photographs, hereafter called images, were taken every second using an intervalometer in aperture priority mode with an f-number of F11 and ISO 400 setup (Table 1). The f-number was set high value for sufficient depth of field so as to obtain sharp images even on rough sea ice surface. The ISO was set to maintain short exposure time preventing image blur.

### 3.2. Preprocessing of Acquired Helicopter-Borne Images

Before acquiring the helicopter-borne VHR images, the time of the camera was synchronized with GPS time. For the acquired images, the acquisition time was matched with the time of 20 Hz GPS logs, and then geographic coordinates, latitude, longitude, and the altitude of the GPS log corresponding to each image were selected if the time difference between the time stamp of the VHR image and the GPS log was less than 0.02 s. Altitude was revised by subtracting 0.8 m to compensate for the position of the GPS logger relative to the camera.

Although geographic coordinates were assigned to each image using high-precision records from the 20 Hz GPS logger (which operated at a higher logging rate than usual GPS loggers, e.g., 1 Hz), the continuous drift of ice floes during the image acquisition causes uncertainties in the geolocations which are used as imaging locations during the mosaicking procedure. Generally, the systematic acquisition of gridded images over a static object, e.g., land, is required for a conventional and precise image mosaicking procedure [35]. However, images acquired over moving objects such as drifting sea ice are allocated to biased geographical coordinates along the motion of the target object or the passage of time.

### 3.3. Compensation of the Effect from Sea Ice Drift in Imaging Locations

Prior to compensating for the effect from sea ice drift, two image subsets, i.e., Subsets I and II, were selected, from relatively lower altitudes of 200 m to 400 m and from altitudes higher than 1000 m, respectively. First acquired images in each subset were designated as reference images for the compensation of the effect of sea ice drift.

To compensate for the effect from continuous sea ice drift, the drift record of the main ice was assessed. The drift trajectory was recorded from the IBRV Araon using an internal 1 Hz GPS logger. After the IBRV Araon’s departure from the main ice on 15 August 2017 around 9:00 UTC, the trajectory of an ice mass balance buoy (IMB) with 1-h GPS logging interval was consecutively used for further comparison of the mosaicked image with the lower-resolution satellite image. The IMB was deployed on 14 August 2017 around 21:00 UTC on the main ice.

The acquisition interval of the imaging location synchronized with the helicopter-borne image was 0.05 s denser than the sea ice drift records from the IBRV Araon, i.e., 1 Hz interval. Therefore, time interpolation was applied to the lower-interval ship data to match the time intervals of the two GPS logs. Before applying time interpolation, the linearity of the drift trajectory was assessed using the heading and trajectory of the IBRV Araon by calculating studentized residuals, i.e., residuals divided by estimated standard errors, from linear regression line [36] to select the appropriate interpolation method. Then linear time interpolation [37,38] was applied if the trajectory was considered to be linear. The studentized residuals were calculated using the geographic coordinates of each record of the trajectory in the Universal Transverse Mercator (UTM) Cartesian coordinate system (Zone 60). The UTM coordinates, locally conformal map projection preserving angles, were measured in meters. Then, it was investigated whether the studentized residuals plotted within the range of ±3. Values larger than three in absolute value were considered as outliers indicating nonlinear motion of the ice floe.

Differences between geographic coordinates of the main ice at the acquisition time of the reference image and at the acquisition time of the other images were calculated based on the time interpolated trajectory of sea ice drift. The differences, i.e., the ice floe motion which occurred during the time gap corresponding to image pairs consisting of the reference image and each other image, were then subtracted from each image except for the reference image. From this compensation of the sea ice drift, the acquisition locations of the images were corrected to be located over the study area, as to be acquired at the same time as the reference image acquisition while maintaining an identical imaging footprint.

### 3.4. Image Mosaicking and Accuracy Assessment

The images with initial and drift compensated geographic coordinates were mosaicked using PhotoScan software (Agisoft LLC, St. Petersburg, Russia) based on the structure-from-motion (SfM) technique [39]. The SfM technique reconstructs three-dimensional structure from partially overlapped images and further allows the orthorectifying and mosaicking of the images. Image mosaicking procedures consisted of: (i) alignment of images; (ii) sparse point detection; (iii) optimization; (iv) dense point construction; (v) digital elevation model (DEM) generation, and (vi) mosaicking orthophotos, as suggested by previous studies (e.g., [40]). We assumed that the SfM technique, without consideration of the helicopter’s inertial motion, produces the mosaicked image of enough geolocation accuracy for a comparison with lower-resolution remote sensing imagery [41].

Accuracy assessment was fulfilled by comparing estimated imaging locations before and after compensation for the effect of sea ice drift for the two image subsets. The estimated imaging location was calculated by determining the relative orientation and location between cameras using the SfM technique [42] during the image mosaicking procedure. For the accuracy assessment, the root-mean-square (RMS) errors for the X coordinate (X error), the Y coordinate (Y error), the X and Y coordinates (XY error), the Z coordinate (Z error), and the X, Y and Z coordinates (total error) for all the cameras were estimated using the following equations [43]:

(1) $$X\;\mathrm{error}\;=\;\sqrt{\sum_{i=1}^{n}{\left(X_{i_{est}}-X_{i_{in}}\right)}^{2}},$$

(2) $$Y\;\mathrm{error}\;=\;\sqrt{\sum_{i=1}^{n}{\left(Y_{i_{est}}-Y_{i_{in}}\right)}^{2}},$$

(3) $$\mathrm{XY}\;\mathrm{error}\;=\;\sqrt{\sum_{i=1}^{n}{\left(X_{i_{est}}-X_{i_{in}}\right)}^{2}+{\left(Y_{i_{est}}-Y_{i_{in}}\right)}^{2}},$$

(4) $$Z\;\mathrm{error}\;=\;\sqrt{\sum_{i=1}^{n}{\left(Z_{i_{est}}-Z_{i_{in}}\right)}^{2}},$$

(5) $$\mathrm{Total}\;\mathrm{error}\;=\;\sqrt{\sum_{i=1}^{n}{\left(X_{i_{est}}-X_{i_{in}}\right)}^{2}+{\left(Y_{i_{est}}-Y_{i_{in}}\right)}^{2}+{\left(Z_{i_{est}}-Z_{i_{in}}\right)}^{2}}$$

where $X_{i_{est}}$ is the estimated value for the X coordinate for the i-th camera position, $X_{i_{in}}$ is the initial value for the X coordinate for the i-th camera position, $Y_{i_{est}}$ is the estimated value for the Y coordinate for the i-th camera position, $Y_{i_{in}}$ is the initial value for the Y coordinate for the i-th camera position, $Z_{i_{est}}$ is the estimated value for the Z coordinate for the i-th camera position, $Z_{i_{in}}$ is the initial value for the Z coordinate for the i-th camera position, and n is the number of camera positions.

The mosaicked image was compared with the SAR image from the TSX satellite to test further feasibility (Table 2). The TSX SAR image was acquired from Airbus Defense and Space for characterization of sea ice in the study area and georeferenced in the UTM coordinate system. The SAR image was converted to a normalized radar cross section (NRCS) with the speckle noise filtering using the refined Lee filter (7 × 7 window) [44]. At the time around the helicopter-borne VHR image acquisition, the study area was covered by clouds. SAR has the merit of being able to acquire images through clouds, and thus the TSX SAR image was considered to be compared with the mosaicked VHR image. Furthermore, the high-resolution SAR image with 3 m spatial resolution is suitable for detailed sea ice studies, e.g., sea ice surface feature detection or sea ice classification [45]. Although the SAR image was acquired on 16 August 2017, after the departure of the IBRV Araon from the main ice, the whole of the main ice was included in the SAR image. While the SAR image was acquired in both HH and VV modes, the HH polarized image was used to visualize the results of the feasibility test.

To test the feasibility of the helicopter-borne mosaicked VHR image for the calibration or validation of lower-resolution remote sensing data, consistency in shape and geographic matching of the main ice included in the mosaicked VHR image with the TSX SAR image was assessed using similarity transformation [47]. The similarity transformation assumes that the shape of an object does not change and considers a combination of translation, rotation and scaling so as to be matched with another object. For applying the similarity transformation, control points were manually and carefully selected from features located inside the main ice. Consequently, the control points were located to avoid regions vulnerable to surface melting or deformation from collisions with other ice floes such as the boundary of the ice floe.

## 4. Results

### 4.1. Results of Helicopter-Borne Image Acquisition 

**Figure 3:**
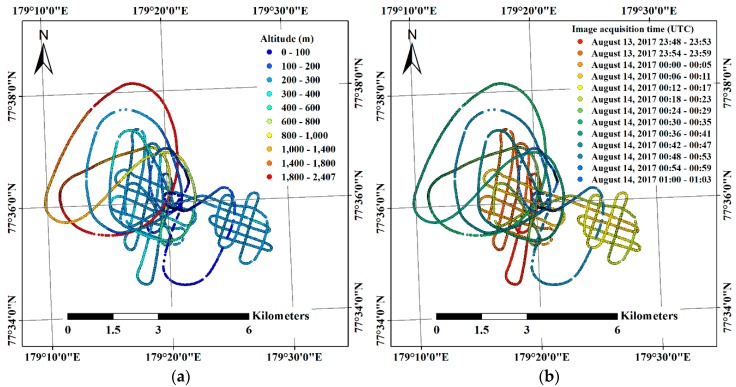
Helicopter-borne VHR image acquisition results: (**a**) locations and altitudes of image acquisition; and (**b**) corresponding image acquisition date and time, expressed as hh:mm.

About 4000 helicopter-borne VHR images were acquired in a single flight of 1 h 14 min 22.45 s during 13–14 August with simultaneous GPS logging (Table 3). The purpose of the flight was not only to obtain VHR images for scientific research purposes, but also for polar bear watching for the safety of the scientists working on the ice floe; therefore, the flight route was not as systematic as in conventional aerial image acquisition over land (Figure 3). Although acquiring images at a 1 s interval was set using an intervalometer, a few shootings were omitted from low temperature and weak endurance of the COTS digital camera to gravitational force acted during turning of the helicopter.

**Figure 4:**
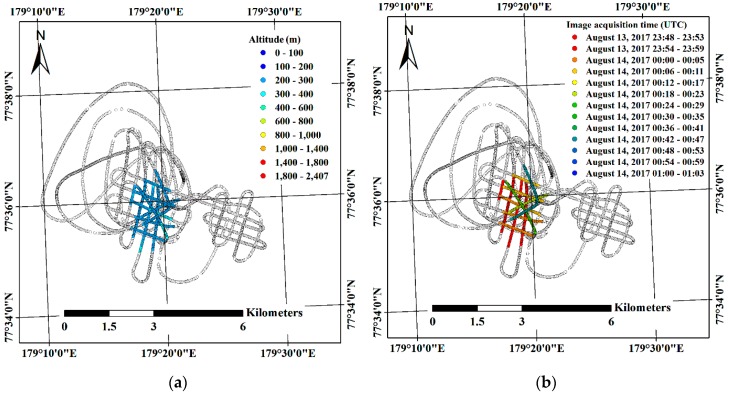

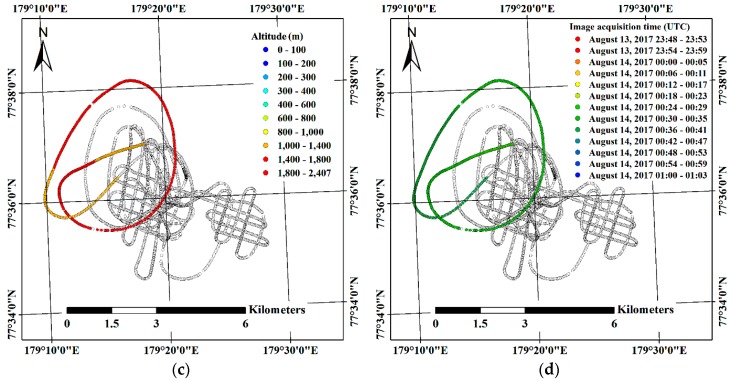
Predefined subsets of acquired VHR images: (**a**) locations and altitudes; and (**b**) acquisition times of the selected images acquired from altitude ranges between 200 and 400 m, designated as Subset I; (**c**) locations and altitudes; and (**d**) acquisition times of the selected images acquired from altitudes higher than 1000 m, designated as Subset II.

As stated in the methods section, two image subsets, i.e., Subsets I and II, were designated from the relatively lower altitude range between 200 m and 400 m and from altitudes higher than 1000 m, respectively (Figure 4). Subset I was comprised of images within a smaller region than Subset II, however the imaging duration, i.e., 55 min 38.75 s, was longer than for Subset II, i.e., 11 min 0 s (Table 4). For the images acquired from altitudes higher than 1000 m, footprints of the images were highly overlapped since the helicopter flew slowly at a relatively higher altitude; thus, half of the images were selected for Subset II by skipping one of two adjacent images. Although the eastern part of the flight route was shown as a grid-like pattern, the images in that part were excluded from grouping the image subsets because the images covered a separate ice floe in which drift trajectory was not available.

### 4.2. Compensation of the Effect from Sea Ice Drift

**Figure 5:**
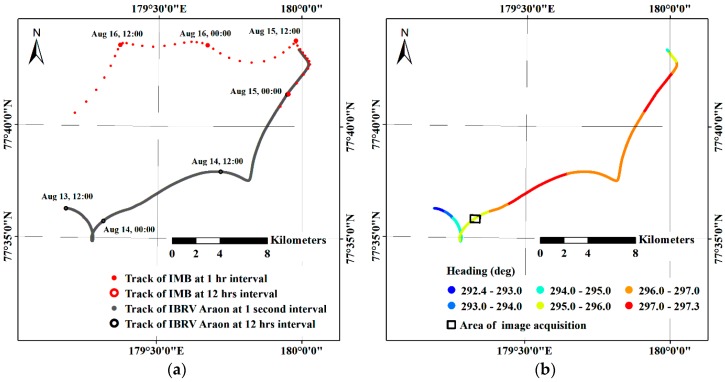

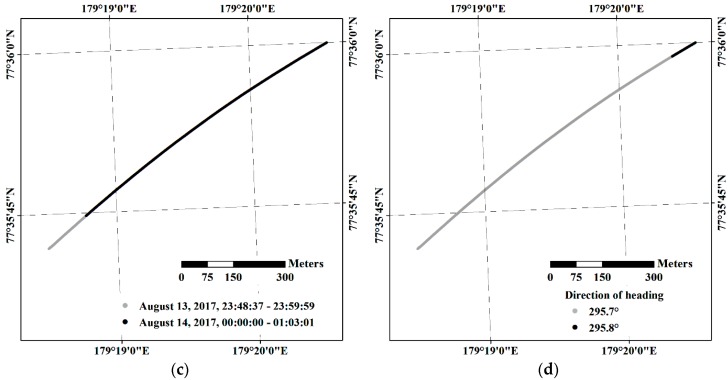
Trajectory of the main ice in the study area: (**a**) an overview of the tracking record from 13 August to 16 August 2017; (**b**) heading information of the IBRV Araon while anchored to the main ice; (**c**) detailed tracking record from the IBRV Araon during the helicopter-borne image acquisition; and (**d**) detailed heading information of the IBRV Araon during the helicopter-borne image acquisition.

The drift record from the IBRV Araon was investigated to compensate for the effect of continuously drifting sea ice (Figure 5). Before applying the time interpolation to the lower-resolution drift records from the ship, the linearity of the drift trajectory was investigated using the heading direction to select a proper interpolation method (Figure 5b,d). During the period of the whole image acquisition, the heading directions of the ship recorded by internal 1 Hz logger showed very low variation, which was evaluated as ±0.0004° (Figure 5d). Thus, we assumed that the main ice drifted without rotation during the period of the image subsets acquisition.

**Figure 6:**
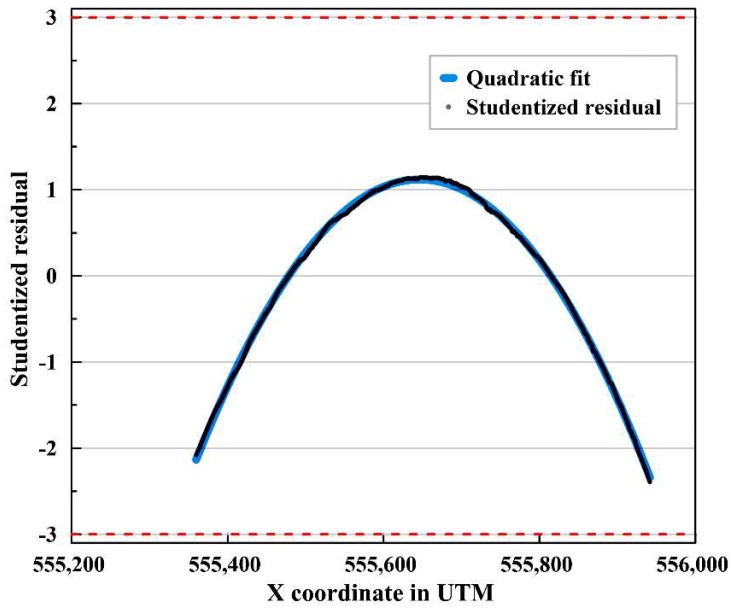
Linearity assessment using studentized residuals of the trajectory of the IBRV Araon during the period of acquisition of the image subsets.

Additionally, the linearity of the ship trajectory during the period when the image subsets were obtained, from 13 August 23:50:39 to 14 August 00:46:19, was assessed using the studentized residuals of each trajectory record. All of the studentized residuals were located within the range between ±3 (Figure 6); therefore, we evaluated that the drift of the main ice was near linear. From these linearity assessment results, linear time interpolation was selected for converting the 1 Hz ship trajectory to be compatible with the images which were time synched with the 20 Hz GPS logs, as it can be assumed that the main ice flow drifted linearly without rotation during the acquisition of the image subsets.

**Figure 7:**
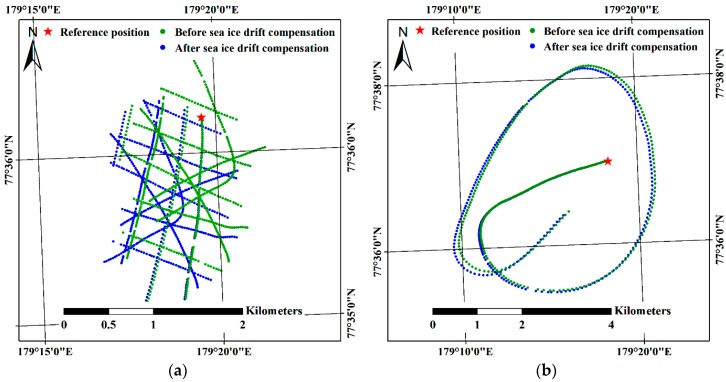
Compensation for the effect of sea ice drift in the helicopter-borne VHR images: (**a**) the imaging locations of Subset I before and after the compensation; and (**b**) the imaging locations of Subset II before and after the compensation.

Among the images comprising Subsets I and II, the first two acquired images of each subset were designated as the reference images for compensation of the effect from sea ice drift with consideration of the linear motion of the main ice without rotation. Differences between geographical coordinates of the main ice at the acquisition time of the reference image and at the acquisition time of the other images were calculated based on the linear time interpolated trajectory of sea ice drift. The location differences were then subtracted from the geographic coordinates of each imaging location; therefore, the imaging locations, except for that of the reference image (which is denoted by red star in Figure 7), were moved after the compensation of the sea ice drift (Figure 7).

### 4.3. Image Mosaicking Results and Evaluation of Errors

**Figure 8:**
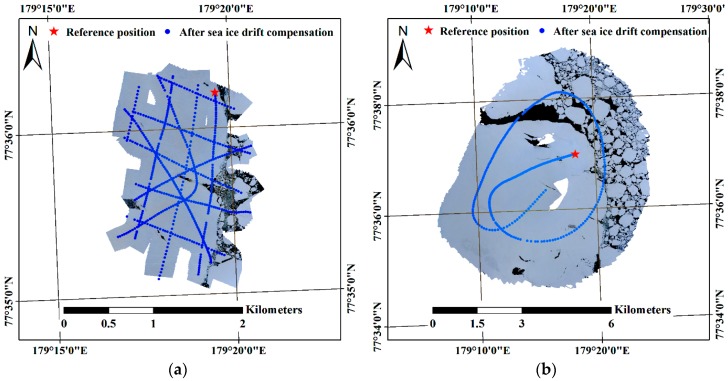
Mosaicked VHR image after compensation for the effect of sea ice drift: (**a**) the mosaicked result using the Subset I images, i.e., those acquired at the altitudes range between 200 and 400 m; and (**b**) the mosaicked result using the Subset II images, i.e., those acquired at altitudes higher than 1000 m.

Mosaicked VHR images from image subsets I and II were generated using the images after compensation for the effect of sea ice drift. The mosaicked VHR images with spatial resolutions of 0.05 m and 0.30 m, respectively, were illustrated with few gaps due to an opportunistic image acquisition approach resulting in partially insufficient overlaps between images (Figure 8). 

**Figure 9:**
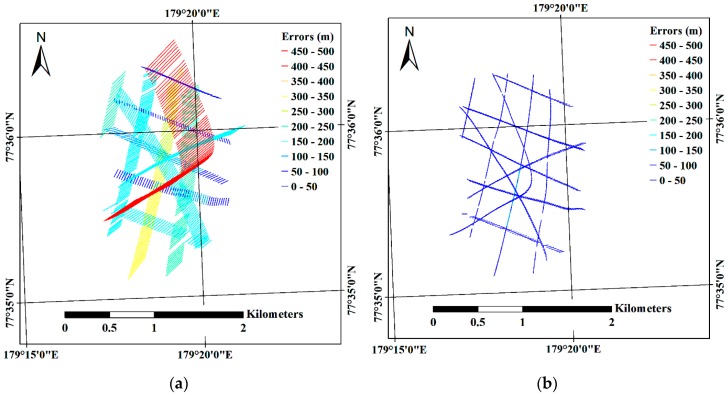

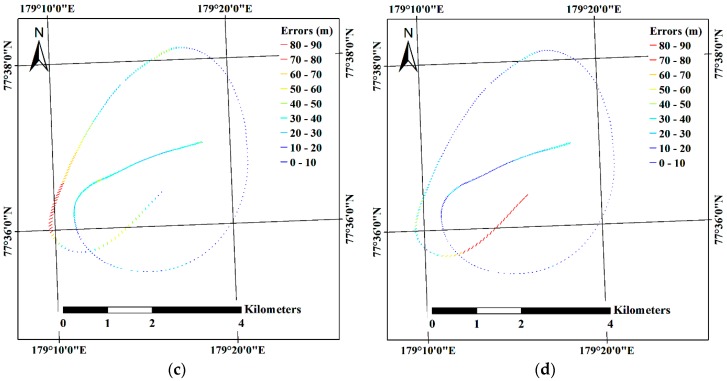
Quality assessment of the imaging locations during the mosaicking processes: (**a**) errors estimated from the imaging locations of Subset I before the sea ice drift compensation; and (**b**) after the sea ice drift compensation; and (**c**) errors estimated from the imaging locations of Subset II before the sea ice drift compensation; and (**d**) after the sea ice drift compensation.

After compensation for the effect of sea ice drift, the location differences between the initial imaging locations and the estimated imaging locations were decreased (Figure 9) for both subsets. Specifically, the X, Y, Z, XZ and total errors for both subsets were reduced after compensation for the effect of sea ice drift (Table 5). The amount of error reduction for Subset I of longer imaging duration (Figure 4b) than Subset II (Figure 4d) was larger than for Subset II. For example, the total error for Subset I was decreased from 241.4 m to 49.9 m, reduced by 79.3% compared with the initial error, whereas the total error of Subset II was decreased from 38.5 m to 29.2 m, reduced by 24.2% compared with the initial error.

### 4.4. Comparison between Helicopter-Borne Mosaicked VHR Image and Satellite SAR Image

To compare the helicopter-borne mosaicked VHR image with the TSX SAR image for a further feasibility test, the mosaicked image needed to be moved along with the drift of the main ice until the timing of the TSX SAR acquisition. Although the entire main ice was included in the SAR image, the SAR image was acquired on 16 August, after the departure of the IBRV Araon from the main ice. Although the trajectory of the ice floe recorded by the IBRV Araon ended after the departure, there were alternative one-hourly records of the trajectory of the same ice floe from the IMB. Thus, it was possible to continuously track the drift.

**Figure 10:**
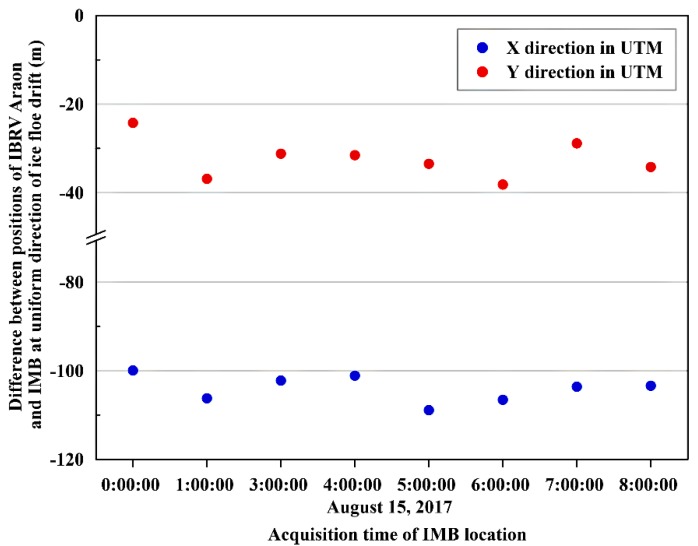
Comparison between the geographic coordinates of trajectories from the IBRV Araon anchored to the main ice and from the ice mass balance buoy (IMB) deployed on the same ice floe, recorded at the time of consistent heading direction.

The usability of the IMB trajectory by combining it with the ship trajectory was assessed by evaluating positional consistency between the two trajectories. The positional difference in the geographic coordinates between the buoy and the ship is maintained as a constant value when the ice floe drifts without rotation. Trajectories recorded at the same time with identical heading direction, i.e., 297.3°, were acquired from 00:00 to 08:00 on 14 August every hour, except for the record at 02:00, when the ship headed to a slightly different direction angle. The selected trajectory records with the identical heading direction varied by ±1.1 m along the X direction and by ±1.6 m along the Y direction (Figure 10). From these results of the assessment of the positional consistency, the continuity of the two trajectories was shown as being maintained from consistent placement of the two logging locations with small variations from intrinsic geolocation errors of the built-in GPS receivers.

**Figure 11:**
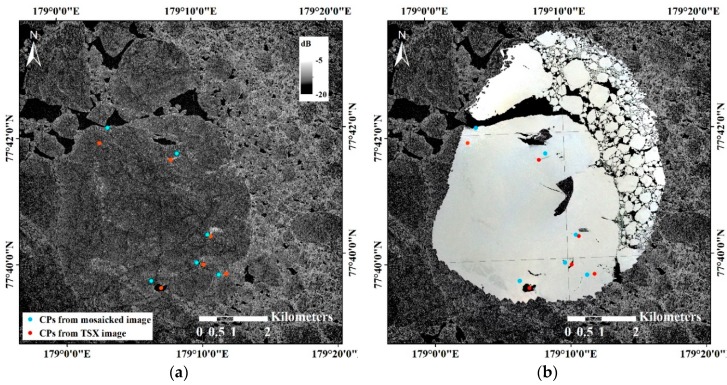

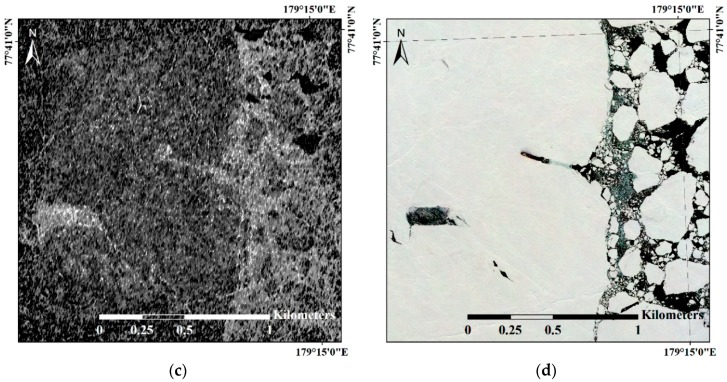
Comparison of the mosaicked helicopter-borne VHR image from Subset II with the TSX SAR image: (**a**) selected control points (CPs) overlaid on the SAR normalized radar cross section (NRCS) image; (**b**) the geographically registered mosaicked image overlaid on the SAR image; (**c**) zoomed area of the SAR image around the IBRV Araon; and (**d**) zoomed area of the mosaicked image around the IBRV Araon.

To test the feasibility of the helicopter-borne mosaicked VHR image for calibration or validation of lower-resolution remote sensing products, consistency in shape and geographic matching with the TSX SAR image were assessed using the similarity transform. The mosaicked image from Subset II with the spatial resolution of 0.30 m was compared with the SAR image since covering a larger area than the mosaicked image from Subset I was more suitable for comparison with the SAR image with a spatial resolution of 3 m. A total of six control points were selected from the surface features. The control points were located inside the main ice to avoid areas prone to surface melting in August (which is belong to sea ice melting season) or to deformation from collisions with adjacent free-drifting ice floes (Figure 11a). In Figure 11a, the red and blue dots indicate the control points selected from the SAR image as the reference points and selected from the mosaicked VHR image before the transformation for conducting geographic matching, respectively. The main ice was precisely registered on the SAR image using the selected control points (Figure 11b–d). During the registration process, the scale and rotation were evaluated as 1.01 and 7.05°, respectively. The RMS sum of all the residuals of the control points was 1.76 m (Table 6).

The distribution of ice floes around the main sea ice was different in the SAR image acquired on 16 August 18:49:52 compared with the mosaicked VHR image comprising images acquired during 14 August 00:27:47.15–00:38:47.15, since the time passage of more than two days caused different drifts and rotations of the adjacent ice floes, and might have caused deformation in boundary regions from collisions between the ice floes.

## 5. Discussion

About 4000 VHR images were opportunistically acquired by using the helicopter carried onboard the icebreaker (Table 3), with the installation of the low-cost COTS digital camera and GPS logger. During a single flight of 1 h 14 min 22.45 s, VHR images covering several kilometers were efficiently acquired (Figure 3), and these were subsequently mosaicked using the SfM technique (Figure 8). Thus, the helicopters carried onboard the icebreaker and the COTS sensors equipped on one of the helicopters were shown to be a simple, cost effective, and practical solution for acquiring a VHR image dataset. This approach can be applicable to other icebreaker polar expeditions.

To compensate for the effect of sea ice drift on the imaging locations, the drift trajectory of the main ice was investigated. Ice floe drift is a complex movement combining translation and rotation; however, during the helicopter-borne VHR image acquisition, the main ice maintained a heading direction with a low variation of ±0.0004° (Figure 5d) and translation was evaluated as near linear; thus, linear time interpolation could be applied for densifying the ship trajectory record. In the case of the trajectory showing nonlinear motion as illustrated before and after the imaging period (Figure 5a), nonlinear time interpolation, e.g., polynomial interpolation, needs to be applied.

After compensating for the effect of sea ice drift, the imaging locations of the two image subsets, excluding the pre-defined reference images, were adjusted as the retrospective locations at the acquisition time of the reference images. This enabled a reduction in the uncertainty in the geolocation of each image without utilizing conventional fixed ground control points (e.g., [48,49]), which are unavailable on continuously drifting sea ice.

The initial errors from the images of Subset I (e.g., the total errors of 241.4 m) were larger than the errors from the images of Subset II (e.g., the total errors of 38.5 m). The multiple discrete flight routes comprising the images of Subset I, which were selected from the altitude range between 200 m and 400 m within the confined area, showed time gaps between each flight route and longer imaging duration, and brought the larger initial errors. Using the images from multiple discrete flight routes like the Subset I images is possibly applicable to generate a mosaicked image in a situation that systematic image acquisition is not available. The errors evaluated using the estimated imaging locations from both image subsets (Subsets I and II) were reduced after the compensation for the effect of sea ice drift (Table 5); the decrease in errors in Subset I was larger than in Subset II as the imaging duration of Subset I, i.e., 55 min 38.75 s, was longer than for Subset II, i.e., 11 min 0 s (Table 4). The extended drifting distance from the longer imaging duration caused increased errors, which can particularly be observed in the bent flight line of Subset I (Figure 9a). The bent flight line was the last flight line among the flight lines of Subset I, with the largest time difference from the reference image, therefore it was more highly affected by the drift of the ice floe than other flight lines. After the compensation for the effect of sea ice drift, the location of the bent line was more adjusted than other flight lines (Figure 7a) and showed a similar amount of errors as other flight lines (Figure 9b). For Subset II, while partial errors were shown to be moved to the latter part of the imaging locations (Figure 9c,d), overall errors were decreased (Table 5). The increased errors in the latter part of the imaging locations might be from the combination of the effects from applying linear time interpolation to the near linear trajectory of sea ice drift, intrinsic errors of the GPS logs assigned to imaging locations, and the helicopter’s inertial motions during turning of the helicopter.

For practical uses, e.g., the calibration or validation of lower-resolution remote sensing data or sea ice products retrieved from the lower-resolution remote sensing data, the mosaicked VHR image needs to be compared directly with the lower-resolution data. The mosaicking processes using the sea ice drift compensated images were fulfilled by using popular commercial SfM software, PhotoScan, and other commercial or open source SfM softwares can be easily applied for generating mosaicked VHR image with well-established image mosaicking routines. The SAR image applied for the feasibility test of direct comparison was acquired on 16 August, after the departure of the IBRV Araon from the main ice. Based on the heading information provided by the IBRV Araon while anchored to the main ice, the ice floe was assumed to be only very lightly rotating during the image acquisition; however, the ice floe was identified to rotate by about 7.05° when compared with the SAR image using the similarity transformation. From this, it can be determined that the rotation occurred after the helicopter-borne VHR image acquisition from continuous drift of the ice floe. More precise direct matching and comparison with the SAR image could be possible if the rotation of the target ice was obtained continuously along the drift using in situ measuring sensors, e.g., multiple sea ice buoys deployed on a single ice floe. 

Although the rotation of the ice floe after the imaging period was not considered, precise registration was fulfilled using a few control points and the similarity transformation with the mosaicked image which was proximally located to the same ice floe in the SAR image by the translation using the combined trajectories of the IBRV Araon and the IMB. In the zoomed area around the IBRV Araon not showing gaps in the mosaicked VHR image (Figure 11c,d), detailed sea ice surface structure, e.g., flat ice surface and pressure ridge, and surface composition, e.g., ice and water, observable in the mosaicked VHR image were directly comparable with the backscatter of the SAR image. The surface characteristics of sea ice can be further studied by comparing both images and can be applied into the calibration or validation of satellite remote sensing-based sea ice products. 

Additionally, the sea ice drift compensated helicopter-borne images can be used for detailed sea ice deformation studies using the DEM or point cloud extracted from the multi-view images. The small-scale topographic features of sea ice (e.g., pressure ridges) can also be extracted by the shadows cast away from the features in the VHR images (e.g., [18]).

## 6. Conclusions

This study proposed two fieldwork-based methods for the practical acquisition of VHR images over drifting Arctic sea ice using low-cost COTS sensors attaching to a helicopter carried onboard an icebreaker, and for compensating for the effect of continuous sea ice drift, which reduces geolocation uncertainty during the image mosaicking procedure. The time interpolated trajectory of the ice floe recorded from an icebreaker was used to compensate for the effect of sea ice drift in each imaging location. After the compensation for the continuous drift, the total errors of the imaging locations were reduced by 79.3% and 24.2% for the two pre-defined image groups, respectively, depending on the imaging duration causing variable drifting distances of individual images. While the ship trajectory was applied to compensate for the effect of sea ice drift in this study, sea ice buoy or other tracking data can be utilized in this purpose.

From the feasibility test of the mosaicked VHR image, performed by comparison with the TSX SAR image, the proposed methods were considered to be suitable for application for direct comparison between lower-resolution satellite remote sensing data and sea ice information from the precisely geolocated VHR ground truth images, from reduced geolocation discrepancy with a simple geographic registration. Under the condition that information of both sea ice motion and rotation are obtained, the geolocation discrepancy will be further reduced. Although helicopter-borne images were used in this study, the proposed methods can also be applicable to mosaic sea ice images obtained from airplane or UAV platforms.

In further study, various properties of sea ice—e.g., surface roughness, distribution of pressure ridge, and surface composition—extracted from precisely georeferenced mosaicked VHR images can be utilized for direct comparison with SAR images and sea ice products extracted from SAR images based on the proposed methods.

## Figures and Tables

**Table 1:** Specifications of the helicopter-borne imaging equipment setup.

Helicopter-Borne Imaging Setup	Specifications
Digital camera	Canon EOS M6
Image acquisition interval	1 s
Imaging mode	Aperture priority mode
Sensor	24 mega-pixel Advanced Photo System type-C (APS-C)
Focal length	22 mm (35 mm equivalent focal length to full frame sensor)
Aperture	F11
Shutter speed	Varies between 1/1000 and 1/3200
ISO	400

**Table 2:** Description of the TerraSAR-X synthetic aperture radar (SAR) image [46] used for comparison with the mosaicked helicopter-borne image.

Satellite Dataset	Specifications
Imaging mode	StripMap
Acquisition date and time	16 August 2017 18:49:52 (UTC)
Centre frequency	9.65 GHz (X band)
Polarization	HH
Spatial resolution	3 m
Swath width	15 km

**Table 3:** Details of the helicopter-borne image acquisition results.

Helicopter-borne VHR Image Acquisition	Specifications
Number of acquired images	4041
Start time of image acquisition	13 August 2017 23:48:37.65 (UTC)
End time of image acquisition	14 August 2017 01:03:00.10 (UTC)
Duration of image acquisition	1 h 14 min 22.45 s
Altitude of imaging location	Up to 2407 m

**Table 4:** Details for image subsets selected from helicopter-borne VHR images.

Image Subset	Number of Images	Imaging Duration
Subset I	664	55 min 38.75 s (13 August 2017 23:50:39.70–14 August 2017 00:46:18.45)
Subset II	324	11 min 0 s (14 August 2017 00:27:47.15–14 August 2017 00:38:47.15)

**Table 5:** Quality assessment using the imaging locations before and after the sea ice drift compensation.

Image Subset	Effect from Sea Ice Drift	X Error (m)	Y Error (m)	XY Error (m)	Z Error (m)	Total Error (m)
Subset I	Before compensation	188.4	150.7	241.2	8.1	241.4
	After compensation	33.5	36.5	49.6	5.5	49.9
Subset II	Before compensation	26.5	24.4	36.0	13.7	38.5
	After compensation	18.9	20.2	27.6	9.4	29.2

**Table 6:** Results of applying the similarity transformation to the mosaicked VHR image using the selected control points (CPs).

No	UTM Coordinates of CPs in Mosaicked VHR Image		UTM Coordinates of CPs in SAR Image		Residuals after Transformation (m)		RMS Error (m)
	X (m E)	Y (m N)	X (m E)	Y (m N)	X (m E)	Y (m N)	
1	550,987.0	8,625,212.1	550,800.3	8,625,026.1	0.0	1.7	1.7
2	551,569.4	8,622,003.6	551,762.8	8,621,946.2	1.3	0.8	1.5
3	552,215.5	8,621,645.7	552,440.4	8,621,672.7	1.2	0.2	1.2
4	551,888.9	8,622,821.1	551,973.2	8,622,784.8	−2.7	−1.9	3.3
5	550,237.3	8,621,463.4	550,518.8	8,621,253.3	−0.5	0.0	0.5
6	548,945.4	8,625,968.4	548,704.8	8,625,518.4	0.6	−0.6	0.9
